# The Role of Mitochondrial Quality Control in Liver Diseases: Dawn of a Therapeutic Era

**DOI:** 10.7150/ijbs.107777

**Published:** 2025-02-10

**Authors:** Jia Li, Wenqin Liu, Jie Zhang, Chao Sun

**Affiliations:** 1 Department of Gastroenterology and Hepatology, Tianjin Medical University General Hospital, Anshan Road 154, Heping District, Tianjin 300052, China.; 2 Department of Gastroenterology, Tianjin Medical University General Hospital Airport Hospital, East Street 6, Tianjin Airport Economic Area, Tianjin 300308, China.

**Keywords:** mitochondrial quality control, liver diseases, reactive oxygen species, mitophagy, mitochondrial dynamics, mtDNA, macrophage heterogeneity

## Abstract

The liver is a vital metabolic organ that detoxifies substances, produces bile, stores nutrients, and regulates versatile metabolic processes. Maintaining normal liver cell function requires the prompt and delicate modulation of mitochondrial quality control (MQC), which encompasses a spectrum of processes such as mitochondrial fission, fusion, biogenesis, and mitophagy. Recent studies have shown that disruptions to this homeostatic status are closely linked to the advent and progression of a variety of acute and chronic liver diseases, including but not limited to alcohol-associated liver disease and metabolic dysfunction-associated fatty liver disease. However, the explicit mechanisms by which mitochondrial dysfunction impacts inflammatory pathways and cell death in the context of liver diseases remain unclear. In this narrative review, we provide a detailed description of MQC, analyze the mechanisms underpinning mitochondrial dysfunction induced by different detrimental insults, and further elucidate how imbalanced/disrupted MQC promotes the progression and aggravation of liver diseases, ultimately shedding light on the mitochondrion-centric therapeutic strategies for these pathophysiological entities.

## Mitochondria and its Function

Mitochondria are double-membrane-bound organelles responsible for a myriad of essential cellular functions, including energy production, glucose and lipid metabolism, cell death, proliferation, and innate immune responses 1. Mitochondria are crucial for cellular energy production, generating approximately 90% of cellular energy through oxidative phosphorylation (OXPHOS), producing adenosine triphosphate (ATP) 2. In brief, energy substrates enter the mitochondrial matrix, where they are processed in the tricarboxylic acid cycle to generate electron carriers (nicotinamide adenine dinucleotide [NADH] and flavin adenine dinucleotide [FADH2]). These electron carriers then transfer electrons through the electron transport chain (ETC), driving the proton pumps to move protons from the matrix to the intermembrane space, thereby establishing an electrochemical gradient, also known as the mitochondrial transmembrane potential 3. The proton gradient is employed by ATP synthase to phosphorylate adenosine diphosphate (ADP) into ATP. Finally, cytochrome c oxidase IV (COX IV), the terminal enzyme of the respiratory chain, accepts electrons from reduced cytochrome c molecules and transfers them to oxygen and protons, producing water as a byproduct 4. Mitochondria are a prime example of endosymbiotic integration, coordinating cellular and organellar signal transduction pathways that are critical for adaptive responses and evolutionary processes. The cornerstone pertinent to this coordination refers to the Mitochondrial Information Processing System, a complex mechanism that interprets signals from ions, proteins, nutrients, and energy states, translating them into targeted genetic programs 5. These programs, in turn, initiate metabolic reprogramming driving key cellular processes such as proliferation, differentiation, contractility, secretion, and apoptosis, all of which are fundamental for organismal survival and evolutionary fitness. However, the precise mechanisms by which these pathways interact within mitochondria to maintain regularly cellular homeostasis remain elusive. In addition to energy production, mitochondria are also involved in other critical cellular functions, including iron metabolism and the regulation of cell proliferation 6. Furthermore, mitochondria play a pivotal role in controlling inflammation and the development of inflammation-related diseases by influencing innate immune responses 7.

## Mitochondrial Quality Control

The MQC mechanism involves the intricate regulation of several processes, including dynamics (fission and fusion), biogenesis, and degradation, all of which are essential to maintain cellular homeostasis 8, 9.

### 1) Mitochondrial Dynamics (fission and fusion)

The fission (division) process is primarily mediated by dynamin-related protein 1 (Drp1), while mitochondrial fusion involves two dynamin-like GTPases, mitofusin 1 (MFN1) and mitofusin 2 (MFN2), as well as optic atrophy 1 (OPA1) and cardiolipin (CL), which mediate the fusion of the outer mitochondrial membrane (OMM) and inner mitochondrial membrane (IMM), respectively 10. Mitochondrial fission separates a single mitochondrion into two smaller fragments, facilitating subsequent degradation, while fusion joins two adjacent mitochondria to orchestrate a larger organelle, preserving mitochondrial DNA (mtDNA) integrity 11-13. Either abnormal mitochondrial fission or fusion has been reported in liver diseases such as non-alcoholic fatty liver disease (NAFLD). Previous studies using mouse models have shown that enhanced mitochondrial fission in hepatocytes is associated with the early stages of NAFLD, and that inhibiting this process can mitigate liver inflammation and fibrogenesis 14-18. The relationship between mitochondrial fusion and the development of NAFLD has been demonstrated in both mice and human models 19-21. Concordantly, the liver of mice and patients with NAFLD presented an increased expression of OPA1 and other proteins involved in mitochondrial function compared to controls. In the case of alcohol-associated liver diseases (ALD), a pronounced hyperactivation of the mitochondrial fission pathway was observed in patients, mice, and cells, characterized by a significant increase in the expression of Drp1 and its adapters/receptors, while the fusion pathway remained unaffected 22-24. As for viral hepatitis, *in vitro* experiments have confirmed that hepatitis B virus (HBV)/hepatitis C virus (HCV)-induced mitochondrial fission may attenuate apoptosis and potentially contribute to persistent viral infection 25, 26.

### 2) Mitochondrial Biogenesis

Mitochondrial biogenesis is under management by certain nuclear transcription factors, including nuclear respiratory factor 1 (NRF1) and nuclear respiratory factor 2 (NRF2), as well as nuclear co-activators such as peroxisome proliferator-activated receptor gamma coactivator 1α (PGC-1α). NRF1 and NRF2 regulate the expression of genes encoding mitochondrial respiratory subunits and mitochondrial transcription factor A (TFAM). TFAM binds to mtDNA at multiple sites, primarily accounting for both mtDNA maintenance and transcription initiation 27. PGC-1α, which does not bind directly to DNA, is recruited to chromatin and acts as a pleiotropic regulator by interacting with nuclear receptors or activating transcription factors, thus promoting mitochondrial biogenesis 28, 29. Mitochondrial biogenesis is crucial for maintaining cellular energy homeostasis and function, and its dysregulation is strongly linked to the pathogenesis of liver diseases, particularly NAFLD. In mice experiencing NAFLD and a well-established NAFLD cell model, the levels of sirtuin 1 (SIRT1) and PGC-1α were reduced, resulting in decreased cell viability, increased apoptosis, lipid accumulation, and reactive oxygen species (ROS) production 30.

### 3) Mitochondrial Degradation

There are multiple pathways involved in regulating MQC, functioning to remove or degrade specific mitochondrial components, portions of mitochondria, or entire mitochondria. The degradation of specific OMM proteins is mediated by the ubiquitin-proteasome system 31, while the degradation of IMM proteins depends on AAA ATPase family proteases, which are located on both sides of the IMM capable of degrading proteins within the matrix and intermembrane space 32. More recently, a novel phenomenon called vesicle derived from the inner mitochondrial membrane (VDIM) has been identified to selectively remove damaged sections of IMM and likely maintains the structure of normal mitochondrial cristae, potentially protecting mitochondria from localized injury 33. A portion of mitochondria can bud off as mitochondria-derived vesicles (MDVs), which serve to transport oxidized proteins to lysosomes or peroxisomes for further degradation 34. Entirely damaged mitochondria can be encapsulated into microvesicles or migrasomes and secreted from cells as extracellular vesicles through processes such as mitocytosis and autophagic secretion of mitochondria 35, 36. Furthermore, damaged mitochondria can be selectively eliminated *via* mitophagy, which involves autophagosomes, or through direct lysosome-mediated micromitophagy. Mitophagy is a well-known process in which excess or damaged mitochondria are selectively removed *via* autophagosomes. These autophagosomes, containing the mitochondria, eventually fuse with lysosomes, where the mitochondria are degraded through either PINK1-PRKN-dependent or -independent mitophagy pathways. Emerging evidence has shown that under certain conditions, mitochondria undergo morphological changes, forming specialized structures such as mitochondrial spheroids and mitochondria-lysosome-related organelles (MLROs), acquiring lysosomal markers for potential degradation. A novel mitochondrial structure observed in murine embryonic fibroblasts involves depolarized mitochondria undergoing morphological remodeling, adopting a ring- or C-shape, and forming ball-like spheroids with an internal lumen surrounded by membranes that contain cytosolic materials 37. MLROs are derived from MDVs that fuse with lysosomes, which is negatively regulated by transcription factor EB (TFEB) and associated with mitochondrial protein degradation 38, 39. Compared to VIDM, MLROs are capable of degrading all mitochondrial components, including the OMM, IMM, and matrix proteins 40. Furthermore, the induction of MLROs is linked to the dedifferentiation of hepatocytes and adipocytes, which can be observed in mouse models of ALD and metabolic-associated fatty liver disease (MAFLD) 38, 41. Hepatocyte dedifferentiation is commonly observed in the late stages of chronic liver diseases and contributes to liver failure, underscoring the potential relevance of MLROs in various liver diseases. Collectively, VDIM, mitochondrial spheroids and MLROs appear to form in response to oxidative mitochondrial damage independently of canonical mechanisms, representing novel mitochondrial dynamics. Since mitophagy is the main pathway of mitochondrial degradation, many studies have confirmed the relationship between mitophagy and liver disease, including NAFLD, ALD and viral hepatitis. Mitochondrial depolarization was observed in hepatocytes, indicating early mitochondrial dysfunction in NAFLD and subsequently triggering mitophagy, affirmative in cell, mouse, and human models 42-44. Furthermore, it has been verified that acute/chronic alcohol exposure and HBV/HCV-induced mitochondrial mitophagy can mitigate hepatic injury and inflammation in multiple models 25, 26, 45-47.

**Figure 1:**
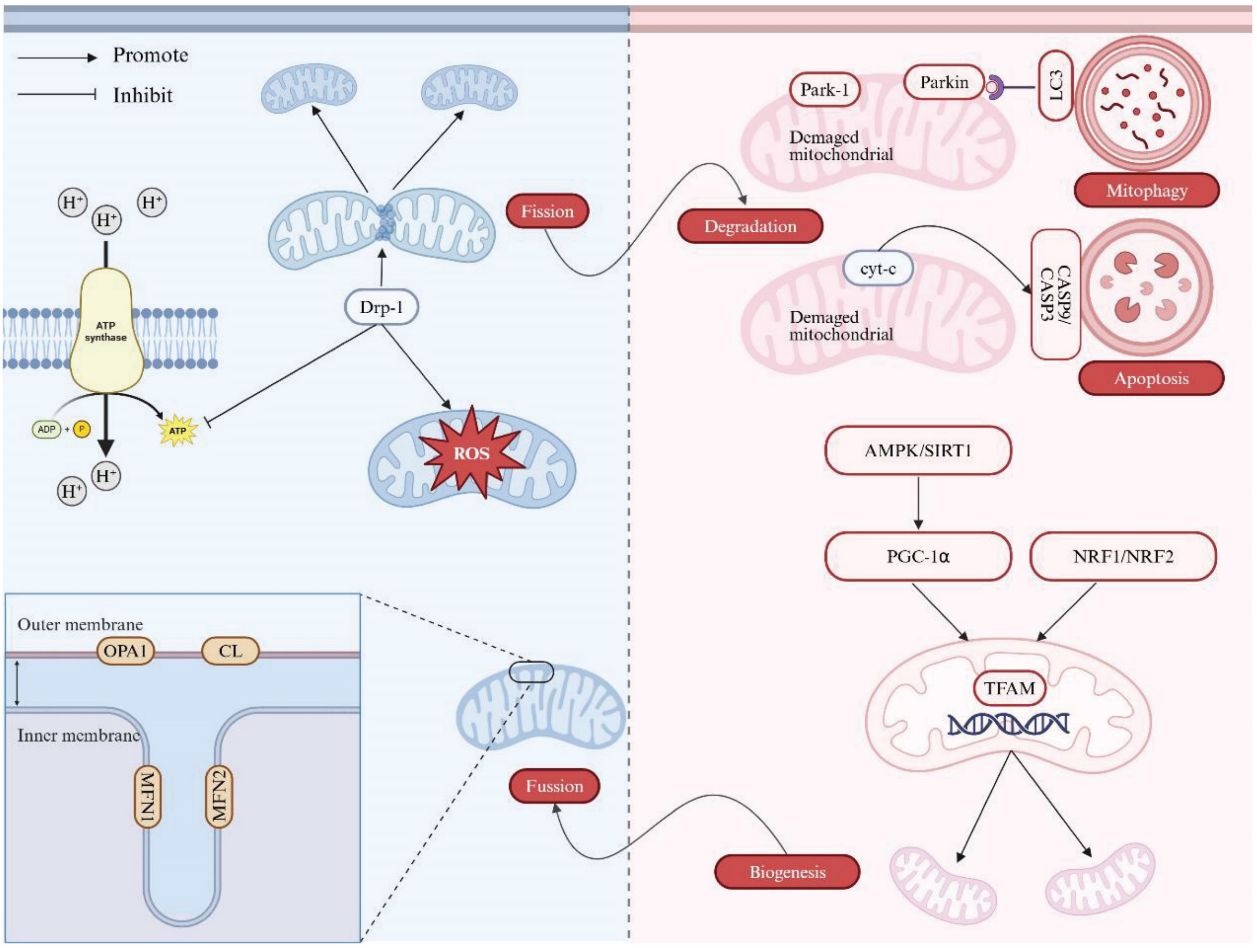
** Regulation of mitochondria quality control.** The mitochondrial quality control mechanism involves mitochondrial fission, fusion, biogenesis, and degradation. (1) Mitochondrial fission and fusion. The fission process is mediated by Drp1, while mitochondrial fusion is regulated by MFN1, MFN2 and OPA1. (2) Mitochondrial biogenesis. Mitochondrial biogenesis is under management by certain nuclear transcription factors, including NRF1 and NRF2, as well as nuclear co-activators such as PGC-1α. (3) Mitochondrial degradation. Mitochondrial can be removed via canonical macro-autophagy and PINK1- PARKIN-mediated mitophagy or other forms of mitochondrial removal. Note: Drp1: dynamin-related protein 1; ROS: reactive oxygen species; OPA1: optic atrophy 1; CL: cardiolipin; MFN1: mitofusin 1; MFN2: mitofusin 2; LC3: light chain 3; Cyt c: cytochrome c; CASP9: caspase 9; CASP3: caspase 3; AMPK: AMP-activated protein kinase; SIRT1: sirtuin1; PGC-1α: peroxisome proliferator-activated receptor gamma coactivator 1α; NRF1: nuclear respiratory factor 1; NRF2: nuclear respiratory factor 2; TFAM: transcription factor A. Created with BioRender.com.

Together, these synergistic actions form a dynamic network that leverages mitochondrial “quality control” and restores homeostasis during energy deprivation or in response to mitochondrial damage. Figure 1 illustrates the regulation of mitochondria quality control. Impaired mitochondrial function and defective quality control mechanisms would contribute to the pathogenesis of various liver diseases, including NAFLD, ALD, and viral hepatitis. Table 1 summarizes the research progress on various types of MQC disorders and their association with hepatopathy.

## Association Between Common Causes of Liver and Diseases Mitochondrial Dysfunction

### Virus Infection

The primary pathogenic mechanism underlying long-term infection on account of HBV involves the induction of mitochondrial dysfunction, including alterations in mitochondrial morphology and dynamics. Ultrastructural analysis of hepatocytes using transmission electron microscopy revealed that HBV infection leads to cytoskeletal disruption and mitochondrial morphological abnormalities, such as the loss of typical tubular or circular shape, disappearance of cristae, and mitochondrial swelling. These changes pertaining to mitochondrial dynamics upregulate Drp-1 expression and promote Parkin translocation, thereby shifting the balance of mitochondrial dynamics toward augmented fission and mitophagy. The enhanced mitochondrial division can reduce ATP production and disrupt calcium flux 48. AMP-activated protein kinase (AMPK), a highly conserved sensor of intracellular adenosine nucleotide levels, is activated in alignment with increased AMP or ADP, which result from decreased ATP production. AMP/ADP binds directly to the γ regulatory subunits of AMPK, causing a conformational change that promotes its activating phosphorylation 49, 50. Additionally, phosphorylation of Thr172 is required for AMPK activation, which is mediated by the serine/threonine kinases or Calcium/Calmodulin-Dependent Protein Kinase Kinase 2 (CAMKK2) in response to AMP/ADP and calcium flux, respectively 51, 52.

**Figure 2:**
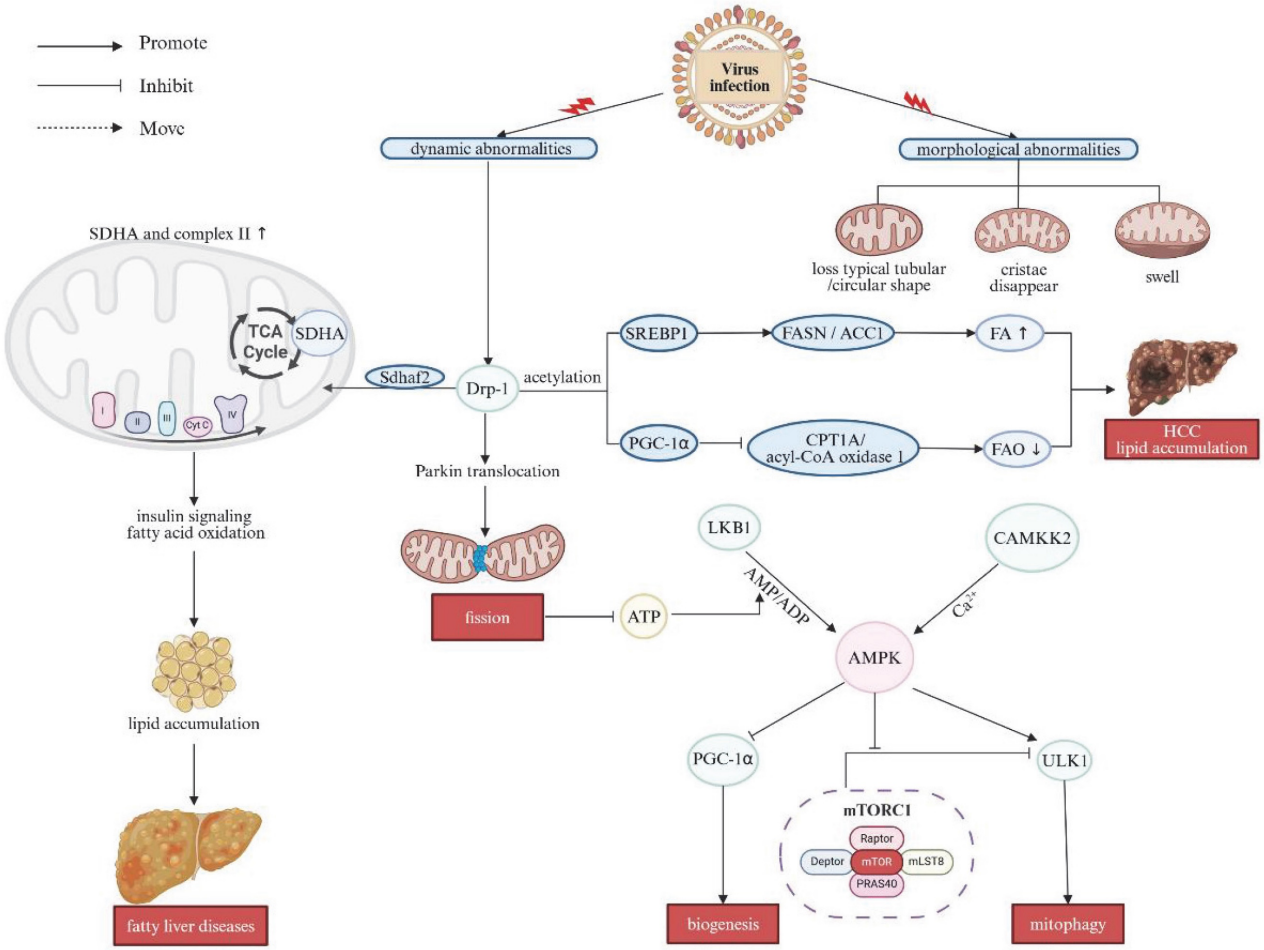
** The effects on mitochondrial quality control after viral infection.** Long-term HBV infection primarily induces mitochondrial dysfunction, including alterations in mitochondrial morphology and dynamics. HBV infection causes cytoskeletal disruption and mitochondrial abnormalities such as the loss of tubular or circular shapes, disappearance of cristae, and mitochondrial swelling. These changes in mitochondrial dynamics upregulate Drp1 expression and promote Parkin translocation, shifting the balance toward increased mitochondrial fission and mitophagy. Drp1 overexpression enhances *de novo* FA synthesis and suppresses FAO by promoting acetylation of SREBP1 and PGC-1α, leading to elevated SREBP1 levels that upregulate lipogenic gene expression, contributing to lipid accumulation in HCC cells. Additionally, overexpressed Drp1 enhances the function of SDHA and complex II in the ETC and TCA cycle, impairing insulin signaling and FAO, further exacerbating fat accumulation. LKB1 and CAMKK2 activate AMPK, stimulating autophagy through a dual mechanism: by activating ULK1 and directly inhibiting the mTORC1 complex's suppressive effect on ULK1. Note: Drp1: dynamin-related protein 1; SDHA: succinate dehydrogenase subunit-A; TCA: trichloroacetic acid; SREBP1: sterol regulatory element binding protein 1; FASN: fatty acid synthase; ACC1: acetyl-CoA carboxylase 1; FA: fatty acid; FAO: fatty acid oxidation; PGC-1α: peroxisome proliferator-activated receptor gamma coactivator 1α; CPT1A: carnitine palmitoyl-transferase 1A; HCC: hepatocellular carcinoma; ATP: adenosine triphosphate; ULK1: UNC-52-like kinase 1; mTORC1: mechanistic target of rapamycin complex 1. Created with BioRender.com.

Once activated, AMPK can stimulate autophagy through a dual mechanism: by activating UNC-52-like kinase 1 (ULK1) alongside directly suppressing the inhibitory effect with regard to the mechanistic target of rapamycin complex 1 (mTORC1) complex on ULK1 53, 54. Furthermore, Ser616-phosphorylated Drp1 forms a complex with autophagosomes marked by Ras-related protein 9 (Rab9) in a ULK1-dependent manner, thereby further triggering autophagic activities 55. This shift contributes to suppress innate immune responses in host cells and facilitates viral replication, giving rising to the persistence of infection. Furthermore, overexpression of Drp1 enhanced *de novo* fatty acid (FA) synthesis and repressed fatty acid oxidation (FAO) by promoting the acetylation of sterol regulatory element binding protein 1 (SREBP1) and PGC-1α. The increase in SREBP1 subsequently upregulated the expression of lipogenic genes, that is, fatty acid synthase (FASN) and acetyl-CoA carboxylase 1 (ACC1), thereby fostering deleterious lipid accumulation in the hepatocellular carcinoma (HCC) cells. Meanwhile, PGC-1α inhibited FAO by downregulating the expression of carnitine palmitoyl-transferase 1A (CPT1A) and acyl-CoA oxidase 1. Therefore, activation of mitochondrial fission significantly accelerated the proliferative alongside metastatic behaviors of HCC cells both in *vitro* and in *vivo* 56. Sdhaf2, a protein essential for the flavination of succinate dehydrogenase subunit-A (SDHA) gene and the assembly of complex II, facilitates mitochondrial localization of this complexes through interaction with Drp1 57. Overexpressed Drp1 also enhances the function of SDHA and complex II in the ETC and the trichloroacetic acid (TCA) cycle, thereby impairing insulin signaling and FAO 58, 59. This perturbation may aggravate the fat accumulation, which is a key pathogenic reason/precipitating factor in various liver diseases 15. These multiple effects on MQC following viral infection are summarized in Figure 2.

### Alcohol Consumption

**Figure 3:**
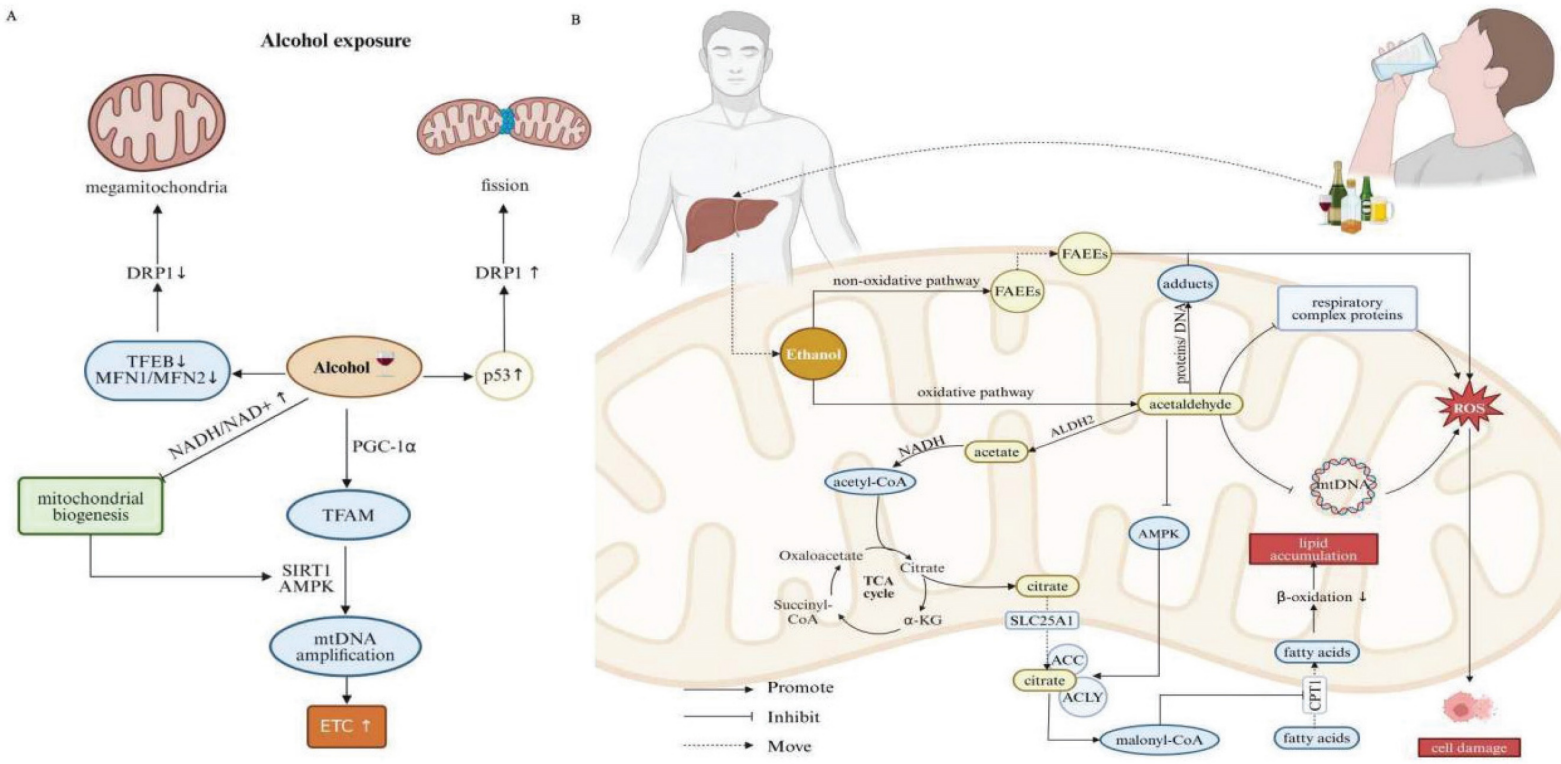
** Relationship between mitochondrial dysfunction and binge drinking.** (A)The effects of alcohol consumption on mitochondrial morphology and dynamics. Alcohol disrupts mitochondrial fission/fusion balance, leading to enlarged mitochondria and fragmentation. It decreases TFEB activation, reducing Drp1 expression and promoting larger mitochondria. Alcohol also activates p53, increasing Drp1 expression and enhancing mitochondrial fission. Chronic alcohol exposure upregulates PGC-1α and TFAM, essential for mitochondrial DNA replication, boosting mitochondrial ETC components and respiration. This process is regulated by upstream factors like SIRT1 and AMPK. However, prolonged binge drinking increases the NADH/NAD+ ratio, reducing SIRT1 and AMPK activity and impairing the PGC-1α pathway, disrupting mitochondrial biogenesis. (B) Alcohol metabolism and its effects on mitochondrial function. Alcohol is metabolized in the liver via oxidative and non-oxidative pathways. In the oxidative pathway, ethanol is first converted to acetaldehyde, which is then metabolized by ALDH2 to acetate. Acetate is further converted to acetyl-CoA, entering the TCA cycle to produce citrate. Citrate is exported to the cytosol via SLC25A1 and converted by ACLY and ACC to malonyl-CoA, which inhibits CPT1 and impairs fatty acid transport into mitochondria, promoting lipid accumulation in hepatocytes. Chronic ethanol consumption inhibits mitochondrial respiratory complex protein synthesis and damages mtDNA, increasing ROS production and causing cell damage. In the non-oxidative pathway, alcohol is converted to FAEEs, destabilizing mitochondrial membranes, disrupting electron flow, and inhibiting OXPHOS. Note: Drp1: dynamin-related protein 1; TFEB: transcription factor EB; MFN1: mitofusin 1; MFN2: mitofusin 2; NADH: nicotinamide adenine dinucleotide; SIRT1: sirtuin 1; AMPK: AMP-activated protein kinase; PGC-1: peroxisome proliferator-activated receptor gamma coactivator 1α; TFAM: transcription factor A; ETC: electron transport chain; FAEEs: fatty acid ethyl esters; ALDH2: acetaldehyde dehydrogenase 2; TCA: trichloroacetic acid; ACC: acetyl-CoA carboxylase; ACLY: ATP citrate lyase; CPT1: CPT1A, carnitine palmitoyl-transferase 1; SLC25A1: mitochondrial citrate carrier. Created with BioRender.com.

Excessive alcohol consumption is a primary and major cause of ALD, a significant global health concern with limited therapeutic options. It is highlighted that alcohol can interrupt mitochondrial function, disrupt ATP production and trigger a burst of oxidative stress, which in consequence culminates in evidently cellular damage and inflammation. Both morphological and functional alterations of mitochondria have been implicated in the pathogenesis of ALD 60, 61. Alcohol impacts the balance between mitochondrial fission/fusion, leading to structural changes such as the formation of megamitochondria and fragmented mitochondria. Increased megamitochondria have been pathologically related to reduced Drp1 expression, arising from decreased TFEB activation 23, 62. Otherwise, alcohol also stimulates p53-mediated induction of Drp1 expression, exacerbating mitochondrial fission and instigating troublesome mitochondrial dysfunction 61. Chronic alcohol exposure initially stimulates mitochondrial biogenesis, resulting in increased expression of components regarding the mitochondrial ETC and enhanced mitochondrial respiration. This response is connected with the upregulation of key factors such as PGC-1α and mitochondrial TFAM, indispensible for mtDNA replication. This process is further dictated by regulators like SIRT1 and AMPK located as upstream cascade, leading to enhanced mitochondrial function. However, prolonged binge disrupts mitochondrial biogenesis by increasing the NADH/NAD+ ratio, which reduces SIRT1 and AMPK activity, impairing the PGC-1α pathway and ultimately giving rise to mitochondrial dysfunction (Figure 3A) 61.

Alcohol is primarily metabolized in the liver through two main pathways: oxidative and non-oxidative manner. While only a small fraction of alcohol undergoes non-oxidative metabolism, it is still threatening to fitness. In particular, alcohol is converted into fatty acid ethyl esters (FAEEs), which can destabilize the mitochondrial membranes and disrupt electron flow in the respiratory chain, ultimately inhibiting OXPHOS 63, 64. The oxidative pathway is the preliminary step by which ethanol is converted into acetaldehyde. Acetaldehyde is a highly reactive metabolite that can form adducts with proteins and DNA, especially within mitochondria, whose extensive accumulation leads to overwhelming oxidative stress and liver injury 63, 65. Damaged mitochondria may exhibit altered redox signaling pathways, further aggravating acetaldehyde-induced oxidative stress and cellular injury. Acetaldehyde is subsequently metabolized by acetaldehyde dehydrogenase 2 (ALDH2), an enzyme predominantly expressed in mitochondria, toward less harmful substance, that is, acetate and NADH. Acetate is then converted into acetyl-CoA, entering the TCA cycle, to produce citrate. Citrate is transported out of the mitochondria into the cytosol through the mitochondrial citrate carrier (SLC25A1), where it undergoes serially enzymatic reactions involving ATP citrate lyase (ACLY) and ACC, responsible for the formation of malonyl-CoA. Malonyl-CoA inhibits carnitine palmitoyl transferase 1 (CPT1), a key enzyme involved in the transport of FAs into the mitochondria. Ethanol exposure also suppresses AMPK activity, which further fosters ACC activity and increases malonyl-CoA levels. Intriguingly, ethanol enhances the sensitivity of CPT1 to malonyl-CoA 66. As a result, the reduced activity of CPT1 diminishes mitochondrial β-oxidation, promoting *de novo* lipogenesis and contributing to lipid accumulation in hepatocytes 61. Moreover, chronic ethanol consumption inhibits the synthesis of mitochondrial respiratory complex proteins. Accordingly, resultant reduced capacity concerning OXPHOS, combined with elevated reducing pressure, orchestrate conditions conducive to ROS production 67. MtDNA is particularly vulnerable to oxidative stress due to its proximity to the IMM, known as the primary intracellular source of ROS. Ethanol-induced damage to mtDNA, if not adequately, effectively or promptly repaired, negatively impacts cellular energy metabolism, further augmenting cellular damage (Figure 3B) 68.

Taken together, alcohol impairs mitochondrial FA oxidation and OXPHOS, disproportionating cellular energy and contributing to the development of hepatic steatosis along with relevant cellular damage.

### Obesity and Metabolic Derangement to Liver

**Figure 4:**
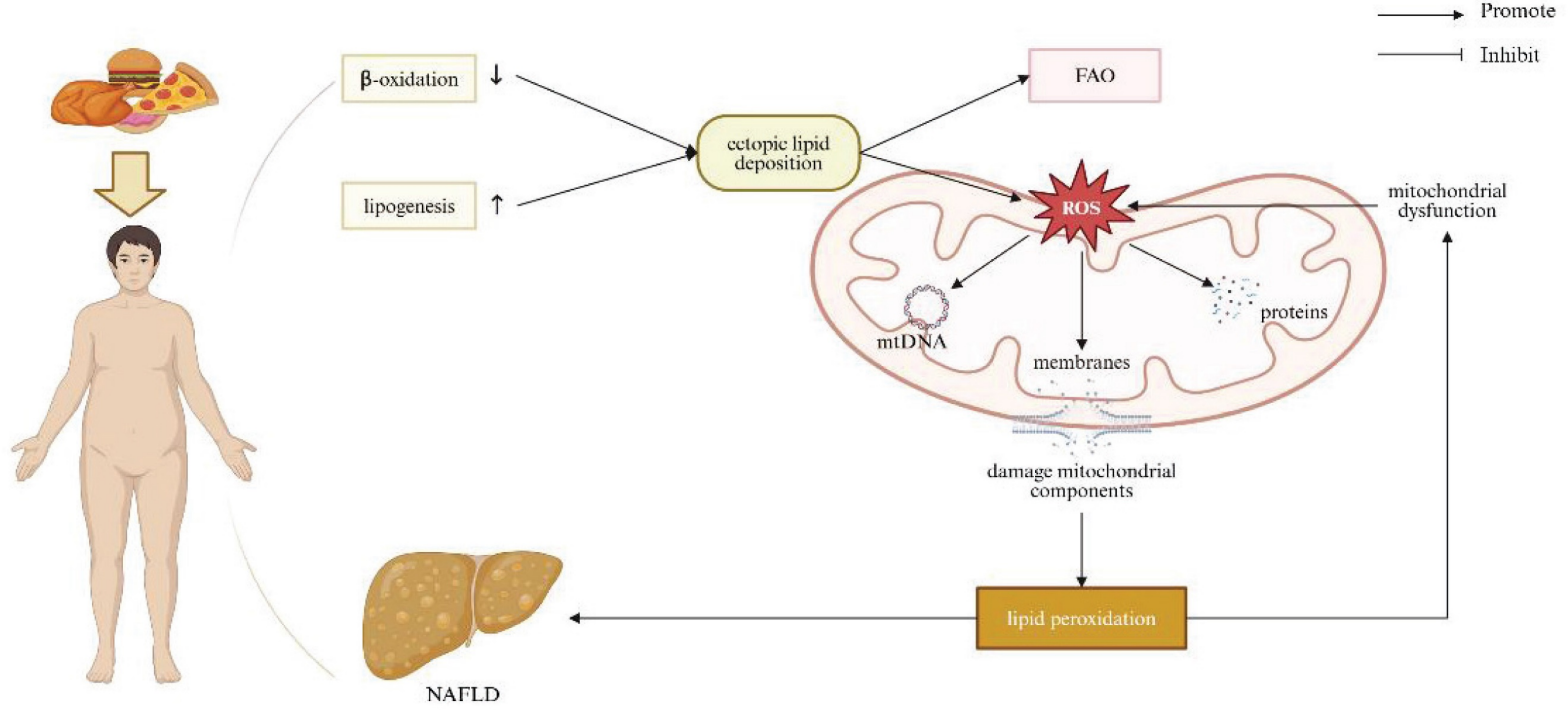
** Relationship between mitochondrial dysfunction and obesity/NAFLD.** Obesity caused by high-fat or high-cholesterol diets disrupts lipid homeostasis, leading to reduced β-oxidation and increased lipogenesis, resulting in lipid accumulation in the liver. Excess hepatocellular lipids stimulate mitochondrial FAO and ROS production. Elevated ROS damages mitochondrial components, including membranes, proteins, and mtDNA, promoting lipid peroxidation and contributing to the development of obesity/NAFLD. Note: FAO: fatty acid oxidation; NAFLD: non-alcoholic fatty liver disease. Created with BioRender.com.

Obesity induced by high-fat or high-cholesterol dietary intakes is associated with dysregulated lipid homeostasis, leading to ectopic lipid deposition in non-adipocyte cells, such as hepatocytes 69. Reduced β-oxidation and increased lipogenesis result in lipid accumulation within haptic parenchyma. Excessive hepatocellular lipids simultaneously stimulate mitochondrial FAO and ROS production. This creates a vicious cycle, wherein damaged mitochondria become dysfunctional, primarily on account of hepatic mitochondrial structural defects and an imbalance in mitochondrial dynamics, indicative of cristae loss, rarefied matrix, reduced levels of OPA-1 and MFN1/2, and increased expression of Drp-1. These alterations impair OXPHOS and further increase ROS production 70. Excessive ROS generation affects mitochondrial components, including membranes, proteins, and mtDNA, and in consequence enhances lipid peroxidation. Cell membranes are significantly susceptible to radical-induced damage due to their abundance pertaining to polyunsaturated FA. ROS-mediated lipid peroxidation occurs in the circumstance of membrane phospholipids interacting with ROS, leading to the oxidation of unsaturated lipid chains and the formation of hydro peroxided lipids and alkyl radicals. This peroxidation negatively impacts membrane structure, compromising their fluidity and integrity 71. A previous study demonstrated that rats challenged by a high-fat diet (HFD) for 6 weeks exhibited changes in mitochondrial oxidative stress markers, including increased hydrogen peroxide production and reduced activity of aconitase and superoxide dismutase 72, 73. Another study consistently indicated that rats exposed to a 14-week HFD exhibited significantly increased serum levels of 8-hidroxy-2-deoxyguanosine in relation to overwhelming oxidative stress systemically 74. Moreover, emerging evidence has implicated that obesity-dictated ROS production can induce DNA damage by repressing the expression of genes involved in DNA repair 75-77. Defective mitophagy has been observed in both *in vivo* and *in vitro* models experiencing NAFLD 78-81. It has been proved that hepatocyte-specific deletion of Parkin exacerbates fatty liver disease and insulin resistance in the context of HFD-fed mice 82. Hepatic mitophagy was reduced in obese mice with fatty liver, uncovering connection between loss of hepatic mitophagy and the advent of NAFLD 83. Collectively, mitochondrial dysfunction exacerbates lipid homeostasis disruption, contributing to the progression of NAFLD (Figure 4).

## The Outcome of Impaired Quality Control of Mitochondrial and Liver Diseases

### Oxidative Stress and Liver Diseases

Mitochondria are essential double-membrane organelles responsible for aerobic respiration in addition to cellular metabolism. When mitochondrial dysfunction occurs, it leads to excessive production of ROS, which is regarded as one of the primary factors contributing to the pathogenesis of various liver injuries 84, 85.

Nuclear factor-kappa B (NF-κB) is a family of transcription factors that plays a central role in inflammatory activities and immunoregulatory actions 86. Under normal conditions, NF-κB is sequestered in the cytoplasm by inhibitor of kappa B (IκB). However, overproduction of ROS triggers the phosphorylation, ubiquitination, and proteasome-mediated degradation of IκB, thereby allowing NF-κB to translocate into the nucleus 87. Nuclear NF-κB is capable of instigating the expression of genes involved in inflammation and survival, such as manganese superoxide dismutase and anti-caspase proteins to exert anti-apoptotic effects. In contrast, sustained elevation of cellular ROS levels can lead to the upregulation of pro-apoptotic cytokines, including interleukin-1β (IL-1β), interleukin-18 (IL-18), and tumor necrosis factor-α (TNF-α), which promote hepatocyte apoptosis and necrosis 7. Moreover, ROS can activate the tumor suppressor protein p53, subsequently, stimulate the expression of Bax, a potent stimulator of mitochondrial pore formation. This process can trigger the mitochondrial permeability transition and facilitate the cell's apoptotic machinery 88. Given the delicate balance between anti- and pro-apoptotic signals, a mild increase in cellular ROS does not necessarily lead to liver cell death, but, excessive, overwhelming and frustrating ROS accumulation can cause liver damage and lesions 89.

Accumulating evidence suggests that defects in the respiratory chain are a key determinant of mitochondrial dysfunction, leading to increased production of ROS 90. Massive ROS generation, in turn, triggers lipid peroxidation, resulting in the formation of malondialdehyde (MDA) and 4-hydroxynonenal (4-HNE). These lipid peroxidation products further inhibit the respiratory chain by disrupting cytochrome c oxidase activity in mitochondrial complex IV, thereby establishing a self-sustaining loop of oxidative stress. Additionally, ROS can damage mtDNA through deletion mutations and influence iron-sulfur (Fe-S) cluster enzymes in the respiratory chain 91. Relative to nuclear DNA, mtDNA is dramatically vulnerable to ROS-induced injury on account of its lack of protective histones in addition to limited and insufficient DNA repairment 92. Extensive mtDNA damage results in the production of defective mitochondrial proteins, compromising the integral respiratory chain and exacerbating oxidative stress 93. Mitochondria-derived ROS further contribute to lipid peroxidation by oxidizing unsaturated lipids. MDA, a byproduct of lipid oxidation, impairs components of the ETC, thereby promoting subsequent ROS generation from the mitochondria 94. The NRF2 is a basic leucine zipper transcription factor that regulates the expression of cytoprotective and antioxidant genes, thereby protecting cells from injurious oxidation induced by inflammation 95. However, excessive ROS production can lead to the downregulation of the NRF2/heme oxygenase-1 (HO-1) signaling pathway, further impairing the cell's antioxidant defense system. On the other hand, mitochondrial ROS production can induce the mitochondrial permeability transition pore (PTP), which increases mitochondrial outer membrane permeabilization. Collectively, aforesaid process facilitates the release of intermembrane space proteins, such as cytochrome c, into the cytosol. Cytochrome c then enhances the translocation of apoptosis-inducing factor (AIF) to the nucleus and activates procaspase-9, forming the apoptosome complex. In this regard, the apoptosome cleaves procaspase-9 into its active form, which subsequently activates effector caspase-3, initiating both apoptotic and necrotic pathways 96.

**Figure 5:**
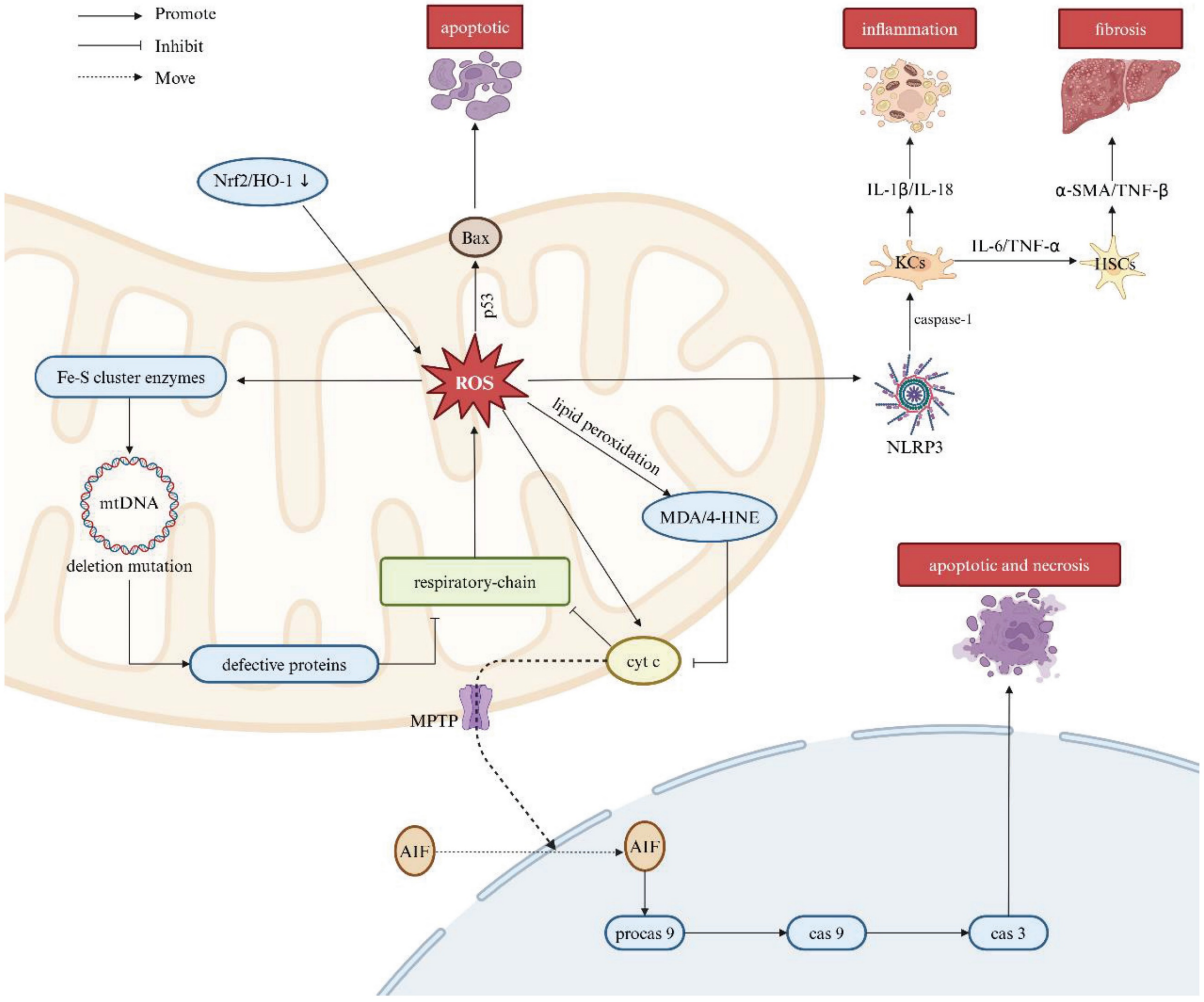
** Association between ROS and liver diseases.** Mitochondrial dysfunction leads to excessive ROS production, a key factor in liver injury pathogenesis. The respiratory chain is central to this dysfunction, driving increased ROS generation. ROS triggers lipid peroxidation, forming MDA and 4-HNE, which impair the respiratory chain by disrupting cytochrome c oxidase in complex IV. This enhances cytochrome c-mediated AIF translocation to the nucleus, activating procaspase-9 and initiating apoptosis. ROS also damage mtDNA, impairing respiratory chain function and exacerbating oxidative stress. Additionally, ROS activate p53, promoting Bax expression, mitochondrial pore formation, and cell apoptosis. ROS activate the NLRP3 inflammasome, leading to proinflammatory cytokine release from KCs, which promotes liver inflammation and fibrosis through HSC activation. Note: ROS: reactive oxygen species; Nrf2: nuclear factor erythroid 2-related factor 2; HO-1: heme oxygenase-1; BAX: BCL-2-associated X protein; Fe-S: iron-sulfur; MDA: malondialdehyde; 4-HNE: 4-hydroxynonenal; MOMP: mitochondrial outer membrane permeabilization; AIF: apoptosis-inducing factor; Procas 9: procaspase-9; Cas 9: caspase-9; Cas 3: caspase-3; NLRP3: NOD-like receptor thermal protein domain associated protein 3; KCs: Kupffer cells; HSCs: hepatic stellate cells; IL-6: interleukin-6; IL-1β: interleukin-1β; IL-18: interleukin-18; TNF-α: tumor necrosis factor-α; TNF-β: tumor necrosis factor-β; α-SMA: alpha-smooth muscle actin. Created with BioRender.com.

Moreover, ROS serve as activators of the NOD-like receptor thermal protein domain associated protein 3 (NLRP3) inflammasome, responsible for the self-cleavage of pro-caspase-1 and activation of Kupffer cells (KCs). Activated KCs release proinflammatory cytokines, including IL-1β and IL-18, thereby aggravating the inflammatory response 97. Furthermore, KCs also secret other cytokines such as IL-6 and TNF-α, which activate hepatic stellate cells (HSCs), consequently promoting collagen production and contributing to the development of liver fibrosis 98, 99. The overproduction of ROS and subsequent damage to mitochondrial proteins and mtDNA can lead to irreversible mitochondrial membrane depolarization and a decrease in mitochondrial membrane potential (Δψm) known as hallmarks pertinent to mitochondrial dysfunction. Under these conditions, protective mitophagy is capable of degrading damaged mitochondria, thereby limiting the release of mtDNA and the production of free radicals. This process also suppresses certain inflammatory response driven by damage-associated molecular patterns (DAMPs) and assists in mitigating secondary liver injury (Figure 5).

### Abnormal Mitophagy and Liver Diseases

Cellular mitophagy is mediated through the Pink1/Parkin, BCL2 interacting protein 3/NIP3-like protein X (BNIP3L/Nix), and FUN14 domain-containing 1 (FUNDC1) pathways, with the Pink1/Parkin pathway being the most extensively studied in mammals 100. In healthy mitochondria, Pink1 is typically undetectable, but it accumulates on the mitochondrial outer membrane in response to membrane depolarization. This accumulation recruits Parkin from the cytosol to specific damaged mitochondria, wherein activating Parkin's E3 ubiquitin ligase. Parkin then ubiquitinates mitochondrial outer membrane proteins, which facilitates the recruitment of autophagy adaptors such as sequestosome 1 (SQSTM1/P62), neighbor of BRCA1 (NBR1) gene, and optineurin. These adaptors bind to the autophagy-associated protein microtubule-associated protein 1 light chain 3 (LC3) at autophagosomes, leading to mitochondrial degradation 101. In addition to Pink1/Parkin, newly discovered mitochondrial outer-membrane proteins, BNIP3 and FUNDC1, also serve as important receptors for hypoxia-induced mitophagy 102, 103. Upon binding to LC3, these proteins enhance the autophagosome formation, in which a bilayer membrane envelops the mitochondrion. The autophagosome then fuses with a lysosome to orchestrate an autophagolysosome, the site for mitochondrion degradation. Prior studies have shown that the regulation of BNIP3 and FUNDC1 is dependent on the stabilization of hypoxia-inducible factor 1-alpha (HIF-1α) 104, 105. However, oxidative stress can disrupt HIF-1α stabilization, impairing HIF-1α-mediated BNIP3/FUNDC1 mitophagy, and thus exacerbating liver injury 106.

Inhibition of mitophagy promotes the cytoplasmic release of mtDNA in macrophages, leading to the activation of the cGAS/stimulator of interferon genes (STING)/NLRP3 signaling pathway. mtDNA can escape from mitochondria across the pores formed by BCL-2-associated X protein (BAX) and BCL-2 antagonist/killer 1 (BAK1), or *via* the mitochondrial permeability transition pore complex (PTPC). This mtDNA leakage activates cyclic guanosine monophosphate-adenosine monophosphate synthase (cGAS), which in turn triggers stimulator of interferon response cGAMP interactor 1 (STING1) signaling and the subsequent production of pro-inflammatory cytokines, including interferon-β (IFN-β), IL-6, and TNF-α. Undoubtfully, these processes may exacerbate hepatic injury and fibrosis 107-109. Moreover, upon release from dysfunctional mitochondria, both mtDNA and ROS can induce secretory IL-1β and IL-18 in the way of inflammasome activation. Additionally, extracellular mtDNA along with other mitochondrial components, such as N-formyl peptides, which accumulate during regulated cell death, can activate and boost neutrophils. This occurs *through* the recognition of mtDNA by Toll-like receptor 9 (TLR9), and N-formyl peptides by the advanced glycosylation end product-specific receptor (AGER) as well as formyl peptide receptor 1 (FPR1), respectively. Finally, extracellular CL, which is typically confined to the IMM, can be presented by dendritic cells on the major histocompatibility complex class I-like molecule CD1d, giving rise to the activation of γδ T cells 109.

**Figure 6:**
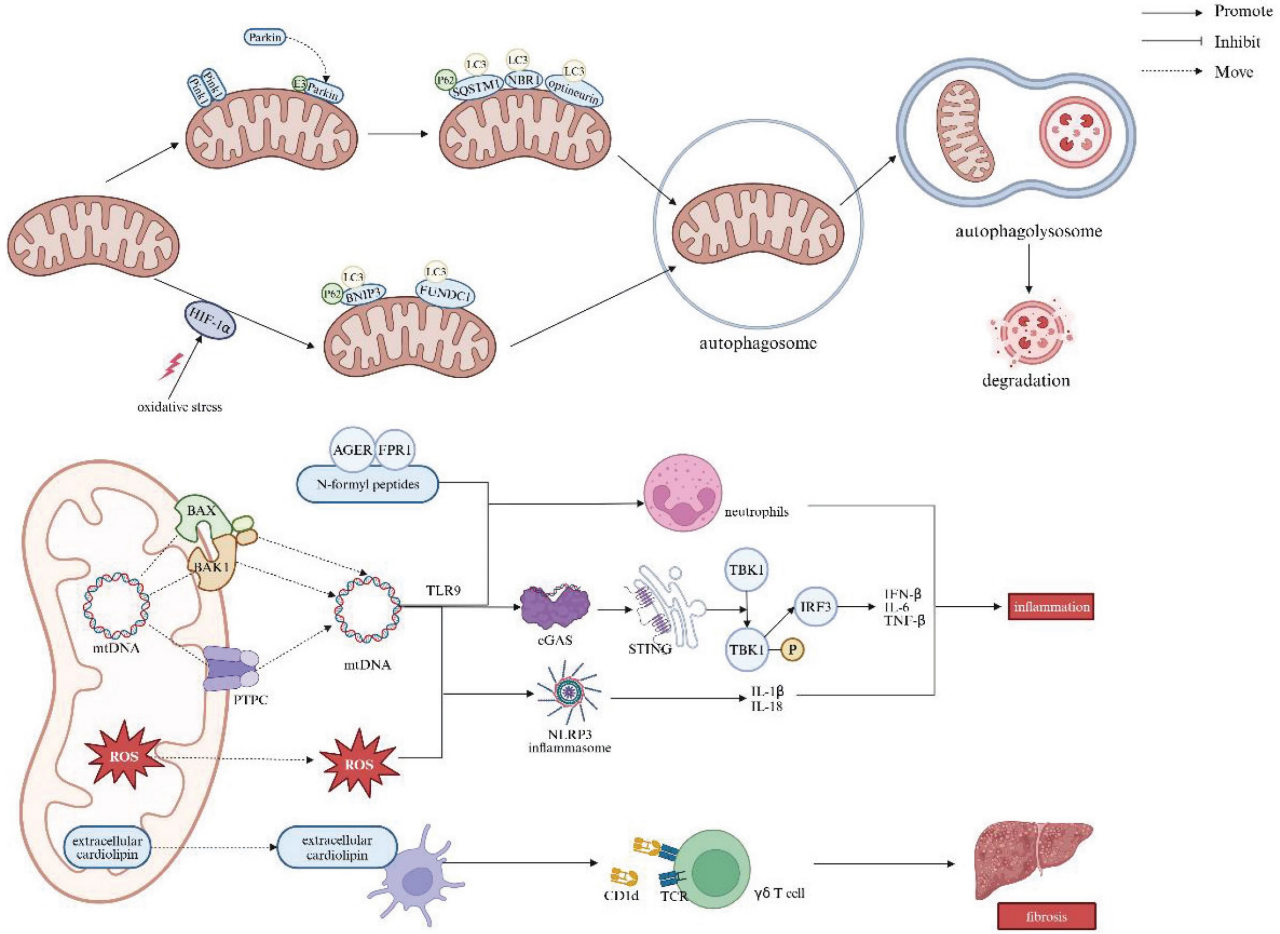
** Relationship between mitophagy and liver diseases.** Mitophagy is mediated by the Pink1/Parkin, BNIP3L/Nix, and FUNDC1 pathways, which promote autophagosome formation and fusion with lysosomes for mitochondrial degradation. Inhibition of mitophagy causes mtDNA release in macrophages, activating the cGAS/STING/NLRP3 pathway. MtDNA leaks through BAX/BAK1 pores or the mitochondrial PTPC, triggering cGAS and STING1 signaling, leading to pro-inflammatory cytokine production (e.g., IFN-β, IL-6, TNF-α). Both mtDNA and ROS activate inflammasomes, inducing IL-1β and IL-18 secretion. N-formyl peptides released during cell death enhance neutrophil activation. Extracellular cardiolipin activates γδ T cells via dendritic cell presentation on CD1d, promoting liver fibrosis. Note: LC3: light chain 3; HIF-1α: hypoxia-inducible factor 1-alpha; BNIP3: BCL2 interacting protein 3; FUNDC1: FUN14 domain-containing 1; BAX: BCL-2-associated X protein; BAK1: BCL-2 antagonist/killer 1; PTPC: permeability transition pore complex; ROS: reactive oxygen species; AGER: advanced glycosylation end product-specific receptor; FPR1: formyl peptide receptor 1; TLR9: Toll-like receptor 9; cGAS: cyclic GMP-AMP synthase; STING: stimulator of interferon response cGAMP interactor; TBK1: TANK-binding kinase 1; IRF3: interferon regulatory factor 3; NLRP3: NOD-like receptor thermal protein domain associated protein 3; TCR: T-cell receptor; IL-6: interleukin-6; IL-1β: interleukin-1β; IL-18: interleukin-18; TNF-β: tumor necrosis factor-β; IFN-β: interferon-β. Created with BioRender.com.

The accumulation of dysfunctional mitochondria crushing the clearance capacity of mitophagy leads to impaired mitophagy, which in turn triggers inflammatory and fibrotic responses by promoting the release of mt-DAMPs and mtDNA. In immune cells, defective autophagy also results in overloaded autophagosomal and autolysosomal contents serving as inflammatory mediators, thereby activating pro-inflammatory signaling pathways. Moreover, mt-DAMPs, including mtDNA, bind to and activate TLR9 on collagen-producing HSCs. In this regard, direct activation of TLR9 subsequently induces HSCs activation and fibrosis (Figure 6) 62.

### Spatial/Temporal Dimension of Macrophage Heterogeneity in Liver Diseases

Liver macrophages, particularly KCs, exhibit significant heterogeneity in response to different stimuli, which can adopt either pro-inflammatory M1-like or anti-inflammatory M2-like phenotypes, depending on the microenvironment and pathological conditions 110. Macrophage polarization, influenced by mitochondrial dynamics, contributes significantly to liver inflammation and fibrosis 111-113. Mitochondrial dysfunction is a key factor in the pathogenesis of liver diseases, especially in macrophages. In the context of liver inflammation, damaged mitochondria in macrophages release mtDNA and ROS, which activate inflammasomes and contribute to the production of pro-inflammatory cytokines. This inflammatory cascade not only exacerbates liver injury but also accelerates the progression of fibrosis 114-116. The relationship between MQC and macrophage heterogeneity further complicates liver disease progression. In NAFLD and liver fibrosis, mitochondrial dysfunction in macrophages can skew the macrophage polarization towards a pro-inflammatory M1 phenotype 111, 117. This polarization amplifies the release of cytokines like TNF-α and IL-1β, which perpetuate liver injury and fibrosis. Conversely, efficient MQC can promote the polarization of macrophages toward an anti-inflammatory M2 phenotype, which aids in tissue repair and resolution of inflammation. Recent studies through single-cell RNA sequencing have revealed that macrophages exhibit a considerably phenotypic diversity than previously established M1/M2 paradigm, underscoring the spatial and time dimension of macrophage heterogeneity in the liver 118. In terms of spatial dimension, the predominant macrophage type in different liver regions may be linked to specific liver diseases. For example, TREM2^+^ CD9^+^ scar-associated macrophages can be observed in human liver cirrhosis exhibiting a profibrogenic gene signature 119, while TREM2^-^expressing macrophages have been identified in the circumstance of liver cancer 120. Moreover, the localization of macrophages in distinct hepatic zones may influence their mitochondrial dynamics, thereby affecting their roles in inflammation and repair. Notably, positioned within liver sinusoids and extending through the space of Disse to hepatocyte layers, KCs are strategically placed to detect changes in the bloodstream and hepatocyte stress, which supports their involvement in immune tolerance and acute responses 121. Additionally, previous studies have addressed that in conditions like NAFLD, there is a shift from a KC-dominant to a monocyte-derived macrophages (MoMFs)-dominant liver macrophage pool, particularly in the portal areas, strongly correlating with disease progression markers 122, 123. Regarding the temporal aspect, liver macrophages exhibit dynamic responses to disruptions in homeostasis, with MoMFs migrating to injury sites to clear debris and initiate tissue repair. While KCs are relatively immobile, chemokine (C-C motif) receptor 2 positive MoMFs can rapidly transit through healthy liver circulation but slow down and accumulate at sites of active inflammation upon injury 124, 125. Thus, the heterogeneity of macrophages in the liver and the critical role of MQC in maintaining macrophage function are central to the development and progression of liver diseases.

## Therapies on Regulating Mitochondrial Quality Control to Protect Liver Diseases

Given mitochondrial function as a cornerstone in the pathogenesis of different liver diseases, therapeutic strategies aimed at regulating MQC held great promise. Here, we discuss potential strategies for therapeutically targeting MQC in liver diseases using small-molecule approaches (Table 2). Mdivi-1, an effective inhibitor of mitochondrial fission, prevented the self-assembly of DRP1 into oligomers and reduced its GTPase activity. In studies conducted by Zhang *et al.* in 2022 and 2024, it was found that Mdivi-1 can inhibit the proinflammatory responses by decreasing the level of phosphorylation of DRP1 at serine 616 site and attenuating the activation of STING signaling in KCs. 126, 127 It is highlighted that AICAR and PXL770 are the agonist of the AMPK pathway. In 2019, Hu *et al.* showed that the gene expression levels of transforming growth factor-β1 (TGF-β1), alpha-smooth muscle actin, and collagen 1 in HSCs are significantly decreased after AICAR treatment in the bile duct ligation mouse model prone to liver fibrosis and portal hypertension. Moreover, AICAR can alleviate portal pressure without changing systemic hemodynamics and alleviate liver cirrhosis in this model. 128 Another investigation conducted by Fouqueray *et al.* revealed that PXL770 can inhibit *de novo* lipogenesis and improve glycemic parameters and indices regarding insulin sensitivity by administering 12 overweight/obese patients with NAFLD and insulin resistance with PXL770. 128 Peroxisome proliferator-activated receptors (PPARs) are ligand-inducible transcription factors that regulate various genes, including PGC-1α 129. Yatsuga *et al.* proved that PPAR agonists, such as Bezafibrate and Thiazolidinediones, enhance mitochondrial biogenesis in the manner of binding to and activating PPARs 130. Idebenone, a structural analogue of coenzyme Q10, exhibits spatially restricted partial agonistic activity towards both PPARα and PPARγ. Tiefenbach *et al.* implementing a mouse model of type 2 diabetes found that idebenone alleviates fatty liver disease by binding to PPAR-α and regulating lipid metabolism 131. Cilostazol, a 2-oxyquinolone derivative, exerts its effects by inhibiting phosphodiesterase III, leading to increased cyclic adenosine monophosphate (cAMP) levels 132. In 2015, Joe *et al.* demonstrated that cilostazol upregulates the expression of PGC-1α, NRF1, and TFAM in hepatocytes, increases COX III/IV expression in addition to mtDNA content, thereby alleviating ischemia/reperfusion-induced hepatic mitochondrial damage 133.

Quercetin, a dietary phytochemical abundant in lots of fruits and vegetables, encompasses anti-inflammatory and antioxidant properties. Zhao *et al.* employed L02 cells to establish an *in vitro* model with ethanol-induced hepatocyte pyroptosis, indicated that quercetin protects hepatocytes by scavenging mitochondrial ROS and promoting PGC-1α-governed mitochondrial homeostasis 134. Urolithin A represents a metabolite of ellagitannin-derived compounds derived from the gastrointestinal microbiota processing. One study reported that UA promotes mitophagy and activates the Nrf2/ARE signaling pathway, consequently, reducing oxidative stress in the context of acetaminophen-induced liver damage 135. Ubiquinol-cytochrome c reductase core protein 2 (UQCRC2) constitutes a component of the mitochondrial ETC. Lu *et al.* implicated that activation of AMPK by metformin restores the expression of UQCRC2 through the activation of the NRF2 pathway, further enhancing Parkin recruitment to the mitochondria and promoting mitophagy, ultimately mitigating liver injury 136. Tetramethylpyrazine (TMP), an extract from *Ligusticum wallichii* Franch, can promotes mitophagy by upregulating UQCRC2 levels, thereby bringing protective effects against alcohol-induced liver injury, inflammation, and ROS overproduction 137. Collectively, these findings suggest that restoring UQCRC2 expression may enhance Parkin-mediated mitophagy, offering potential therapeutic benefits for liver diseases. Tong *et al.* demonstrated that Diisopropylfluorophosphate treatment, an FDA-approved orally active iron chelator 138, inhibits the ferroptosis markers ACSL4 and ALOX15 in MAFLD mouse models, while ameliorates hepatic inflammation *via* the induction of mitophagy 139. In 2022, Yu *et al.* found that icaritin and doxorubicin remodel the immunosuppressive tumor microenvironment and trigger robust immune memory response, efficiently improving anti-HCC effect at an early stage in mouse HCC model. Their findings unveil an anti-HCC mechanism concerning icaritin on mitophagy and provide an effective immune-based therapeutic strategy in the context of HCC 140. Overall, pharmacological targeting MQC shows promise as a potential treatment for liver diseases, although many studies are still at the animal or cell model stage.

## Conclusion

MQC plays a crucial role in maintaining liver health and preventing the onset and progression of liver diseases. Understanding the intricate mechanisms with respect to MQC across distinct pathophysiological conditions undisputedly opens up new avenues for therapeutic interventions. Future researches focusing on mitochondrial-targeted treatments could offer novel strategies to improve the prevention and management of liver diseases, in hopes of achieving better clinical outcome.

## Figures and Tables

**Table 1:** The association between various types of mitochondrial quality control disorders and liver diseases

Type	Liver Diseases	Species	Study Protocol	Outcomes	Reference
Fission	NAFLD	Mice	Mice were fed a standard diet or choline-deficient, L-amino acid-defined diet with vehicle or mitochondrial division inhibitor-1.	Mitochondrial fission increase in hepatocytes was linked to early-stage NASH, including steatosis, liver inflammation, and hepatocyte ballooning.	14
	NASH	Mice	NASH mice were fed an MCD diet for 12 weeks to induce severe NASH, while control mice received a standard diet.	Compared to the control group, NASH liver organoids exhibited abnormal mitochondrial and organoid morphology, along with elevated levels of DRP1, mitochondrial mitogen protein, and ROS production.	15
	ALD	Mice/ VL-17A cell	Wild-type (WT) mice and liver-specific Drp1 knockout (KO) mice were divided into two groups and fed either a control diet or an ethanol diet. The human hepatoma VL-17A cell was treated with or without 100 mmol/L ethanol for 3 or 14 days.	Ethanol-fed mice exhibited exacerbated hepatic megamitochondria compared to wild-type mice on the same diet, while also showing reduced ethanol-induced toxicity. Prolonged ethanol exposure in VL-17A cells reduced Drp1 translocation, whereas Drp1 inactivation in the absence of ethanol resulted in hyperfused megamitochondria.	23
	AH	Human	Hepatic expression of mitochondria-shaping proteins (MSPs) was assessed in alcoholic hepatitis (AH) patients using DNA microarray (n = 15) and RT-PCR (n = 32). Drp1 activation was studied in mitochondria from ALD patient liver biopsies (n = 8). Alcohol effects on mitochondrial dynamics and MSP expression were examined in precision-cut liver slices (PCLS) exposed to ethanol (50-250 mM) for 24 hours.	AH patients exhibited hyperactivation of the fragmentation pathway, with increased Drp1 and its adapters/receptors. Drp1 translocation to mitochondria was induced in severe ALD patients and mildly affected in PCLS after short-term EtOH exposure.	22
Fusion	NAFLD	Human	By employing a multimodal imaging approach, the structure and morphometric parameters of normal and giant mitochondria were analyzed in four patients.	Giant mitochondria, resulting from mitochondrial fusion, exhibit impaired function due to a reduced surface area-to-volume ratio and disorganization of the inner and cristae membranes.	21
	NAFLD	Mice/ Human	Male mice selectively lacking OPA1 were fed a high-fat diet for 20 weeks. The human transcriptomic data were retrieved from the gene expression omnibus (GEO) repository.	Mice lacking OPA1 were protected from hepatic steatosis and obesity due to reduced lipid absorption. The liver of NAFLD subjects showed increased OPA1 expression and elevated levels of proteins involved in mitochondrial function compared to controls.	19
Biogenesis	NAFLD	Mice/ HepG2 cell	Six-week-old mice were randomly assigned to the control (ordinary diet) or HFD group (high-fat diet) for 8 weeks, with eight animals per group. HepG2 cells were treated with 1.5 mM oleic acid (OA) for 48 hours to induce NAFLD in the cell model, and SIRT-1 induction was monitored.	In mice with NAFLD, the levels of SIRT-1 and PGC-1α were reduced. In a well-established NAFLD cell model, exposure of the HepG2 hepatocyte cell line to OA led to decreased cell viability, increased apoptosis, lipid accumulation, and reactive oxygen species production.	30
	ALD	Mice	Mice were divided into two groups: intragastric alcohol-fed and control. They were fed a high-fat diet, with or without alcohol, starting at 22.7 g/kg/day and gradually increasing to 38.4% of total caloric intake after 4 weeks.	Intragastric alcohol feeding in mice led to 1) altered mitochondrial morphology with increased elongated mitochondria and 2) enhanced mitochondrial biogenesis in the liver, associated with upregulation of PGC-1α.	141
Degradation	NAFLD	Mice/AML 12 cell	Eight-week-old male Nrf2+/+ (WT) and Nrf2-/- mice were randomly assigned to the Ctrl, sulforaphane (SF), HFD, or HFD + SF groups. AML12 cells were treated with the indicated concentration of mixed fatty acid (FA), palmitic acid (PA), stearic acid (SA), palmitoleic acid (PO), or oleic acid for 24h.	HFD increased KEAP1 and decreased NRF2 in the liver of Nrf2+/+ mice, while altering autophagy-related proteins in an NRF2-independent manner. Autophagosome formation was reduced in both HFD mouse liver tissue and FA-treated AML12 cells. HFD and SFAs (FA, PA, SA), but not MUFAs (PO, OA), suppressed autophagosome biogenesis.	42
	NASH	Mice	C57BL/6 mice were fed a Western diet (high fat, fructose and cholesterol) for 2 weeks, 2 months and 6 months.	After 2 weeks on a Western diet, mitochondrial depolarization (mtDepo) was observed in 50-70% of hepatocytes, indicating early mitochondrial dysfunction in NASH, which triggered mitophagy and increased mitophagic markers at 2 and 6 months, while concurrently impairing autophagic processing.	43
	NASH	Human	Liver samples were collected from 130 adults with clinical obesity undergoing bariatric surgery at the University of Missouri Hospital, grouped into No disease/control (CTRL, n = 13), Nonalcoholic fatty liver (NAFL, n = 34), Borderline-NASH (B-NASH, n = 27), and Definite-NASH (D-NASH, n = 56).	Hepatic markers of macro-autophagy, including ULK1, were significantly lower in D-NASH compared to CTRL.	44
	ALD	Mice	The WT and Parkin KO mice were treated with alcohol by the acute-binge and Gao-binge models.	Parkin KO mice exhibited reduced mitophagy, β-oxidation, mitochondrial respiration, and cytochrome c oxidase activity following acute alcohol treatment, compared to WT mice.	142
	CHB/ CHC/ NASH	Human	Patients with chronic hepatitis B (CHB; n = 146) were compared to patients with chronic hepatitis C (CHC; n = 33), NASH (n = 12), and healthy controls (n = 24).	PRKN mRNA was significantly increased in patients with CHB, CHC, or NASH compared to controls.	47
Fission and Mitophagy	HCV infection	Huh7 cell	Huh7 cells were cultured, and cell culture-derived HCV of JC1 and JFH1 strains were used.	HCV-induced mitochondrial fission and mitophagy serve to attenuate apoptosis.	25
	HBV infection	Huh7 cell/ HepG2 cell/ HepAD38 cell	Cells were cultured under appropriate conditions and transfected with siRNA for further study.	HBV and its HBx protein promote mitochondrial fragmentation and mitophagy by inducing Drp1 expression and Parkin translocation.	26

Note: NAFLD: nonalcoholic fatty liver disease; NASH: nonalcoholic steatohepatitis; MCD: methionine- and choline deficient diet; Drp1: dynamin-related protein 1; ROS: reactive oxygen species; ALD: alcohol-associated liver diseases; WT: wild-type; KO: knockout; AH: alcoholic hepatitis; MSPs: mitochondria-shaping proteins; PCLS: precision-cut liver slices; HFD: high-fat-diet; OA: oleic acid; SIRT-1: sirtuin 1; PGC-1α: proliferator-activated receptor gamma coactivator 1α; OPA1: optic atrophy 1; GEO: gene expression omnibus; SF: sulforaphane; FA: fatty acid; PA: palmitic acid; SA: stearic acid; PO: palmitoleic acid; KEAP1: kelch-like ECH-associated protein 1; NRF2: nuclear factor erythroid 2-related factor 2; SFAs: saturated fatty acids; MUFAs: monounsaturated fatty acids; mtDepo: mitochondrial depolarization; CTRL: no disease/control; NAFL: nonalcoholic fatty liver; B-NASH: borderline-NASH; D-NASH: definite-NASH; ULK1: UNC-52-like kinase 1; CHB: chronic hepatitis B; CHC: chronic hepatitis C; PRKN: parkinson juvenile disease protein; HCV: hepatitis C virus; HBV: hepatitis B virus; Hbx: hepatitis B virus X protein.

**Table 2:** Pharmacological targeting of mitochondrial quality control

Target Type	Molecule Name	Functional Mechanism	Model			Clinical Trial		
			Species	Period	Outcome	Status	Period	Identifier
Fission	Mdivi-1	Inhibit the activation of DRP1 signaling	Mouse 127	8 weeks	Reduced hepatic steatosis			
Biogenesis	AICAR	Stimulate AMPK	Rat 128	2 weeks	Decreased hepatic fibrogenesis			
	PXL770	Stimulate AMPK				Phase Ⅰ /Completed	5 weeks	NCT05441904
	Bezafibrate /Thiazolidinediones	Bind to and activate PPARs	Mouse 130	6 weeks	Improved all liver functions and reduced oxidative stress	PhaseⅢ /Completed	24 months	NCT01654731
	Idebenone	Bind with PPAR-α	Mouse 131	3 weeks	Reversed fatty liver development	PhaseⅡ /Active	48 weeks	NCT04669158
	Cilostazol	Increase the expression of PGC-1α and the content of mtDNA	Mouse 133	12 weeks	Reduced liver lipid accumulation	PhaseⅡ /Active	12 weeks	NCT04761848
	Quercetin	Scavenge mitochondrial ROS and promote PGC-1α-mediated biogenesis	L02 cell 134	24 hours	Downregulated redox status, lipid droplets, and LPO release. Restored damaged mitochondrial membrane potential, repaired mtDNA damage, and improved mitochondrial dynamics	Phase I /Completed	4 weeks	NCT01438320
	Urolithin A	Promote mitophagy and activate Nrf2/ARE signaling	Mouse 135	48 hours	Reduced oxidative stress	Phase II /Completed	4 Months	NCT03283462
Mitophagy	Metformin	Activate AMPK and restore the expression of UQCRC2	Mouse 136	10 days	Reduced ethanol-induced liver injury	Phase Ⅳ /Completed	48 weeks	NCT00546442
	TMP	Restore UQCRC2 expression	Mouse 137	4 weeks	Decreased oxidative stress and hepatic inflammation			
	Diisopropylfluorophosphate (DFP)	Induce mitophagy by iron chelation	Mouse 139	2 weeks	Decreased hepatic inflammation	Phase Ⅳ /Completed		NCT01767103
	Icaritin	Recover Δψm and inhibit mTOR complex I	Mouse 140	1 week	Prolonged survival time of mice at the advanced stage of HCC	Phase Ⅱ /Completed		NCT01972672

Note: Drp1: dynamin-related protein 1; AICAR: 5-Aminoimidazole-4-carboxamide 1-β-D-ribofuranoside; AMPK: AMP-activated protein kinase; PPARs: peroxisome proliferator-activated receptors; PGC-1α: peroxisome proliferator-activated receptor gamma coactivator 1α; mtDNA: mitochondrial DNA; ROS: reactive oxygen species; LPO: lipid peroxidation; Nrf2: nuclear factor erythroid 2-related factor 2; ARE: antioxidant response element; TMP: tetramethylpyrazine; UQCRC2: ubiquinol-cytochrome c reductase core protein 2; mTOR: mechanistic target of rapamycin; HCC: hepatocellular carcinoma.

## Abbreviations

ACC: acetyl-CoA Carboxylase
ACC1: acetyl-CoA carboxylase 1
ADP: adenosine diphosphate
AGER: advanced glycosylation end product-specific receptor
AIF: apoptosis-inducing factor
ALD: alcohol-associated liver diseases
ALDH2: acetaldehyde dehydrogenase 2
AMPK: AMP-activated protein kinase
ATP: adenosine triphosphate
BAK1: BCL-2 antagonist/killer 1
BAX: BCL-2-associated X protein
BNIP3L: BCL2 interacting protein 3
cAMP: cyclic adenosine monophosphate
CAMKK2: calcium/calmodulin-dependent protein kinase kinase 2
cGAS: cyclic guanosine monophosphate-adenosine monophosphate synthase
CL: cardiolipin
COX IV: cytochrome c oxidase IV
CPT1: carnitine palmitoyl transferase 1
CPT1A: carnitine palmitoyl-transferase 1A
DAMPs: damage-associated molecular patterns
Drp1: dynamin-related protein 1
ETC: electron transport chain
FA: fatty acid
FADH2: flavin adenine dinucleotide
FAEEs: fatty acid ethyl esters
FAO: fatty acid oxidation
FASN: fatty acid synthase
Fe-S: Iron-sulfur
FPR1: formyl peptide receptor 1
FUNDC1: FUN14 domain-containing 1
HBV: hepatitis B virus
HCC: hepatocellular carcinoma
HFD: high-fat diet
HIF-1α: hypoxia-inducible factor 1-alpha
4-HNE: 4-hydroxynonenal
HO-1: heme oxygenase-1
HSCs: hepatic stellate cells
IFN-β: interferon-β
IL-1β: interleukin-1β
IL-18: interleukin-18
IMM: inner mitochondrial membrane
IκB: inhibitor of kappa B
KCs: Kupffer cells
LC3: light chain 3
MAFLD: metabolic-associated fatty liver disease
MDA: malondialdehyde
MDVs: mitochondria-derived vesicles
MFN1: mitofusin 1
MFN2: mitofusin 2
MLROs: mitochondria-lysosome-related organelles
MoMFs: monocyte-derived macrophages
MQC: mitochondrial quality control
mtDNA: mitochondrial DNA
mTORC1: mechanistic target of rapamycin complex 1
NADH: nicotinamide adenine dinucleotide
NAFLD: non-alcoholic fatty liver disease
NBR1: neighbor of BRCA1
NF-κB: nuclear factor-kappa B
NLRP3: NOD-like receptor thermal protein domain associated protein 3
Nix: NIP3-like protein X
NRF1: nuclear respiratory factor 1
NRF2: nuclear respiratory factor 2
OMM: outer mitochondrial membrane
OPA1: optic atrophy 1
OXPHOS: oxidative phosphorylation
PGC-1α: peroxisome proliferator-activated receptor gamma coactivator 1α
PPARs: peroxisome proliferators-activated receptors
PTP: permeability transition pore
PTPC: permeability transition pore complex
Rab9: ras-related protein 9
ROS: reactive oxygen species
SDHA: ruccinate dehydrogenase subunit-A
SIRT1: sirtuin 1
SLC25A1: mitochondrial citrate carrier
SQSTM1: sequestosome 1
SREBP1: sterol regulatory element binding protein 1
STING: stimulator of interferon genes
TCA: trichloroacetic acid
TFAM: transcription factor A
TFEB: transcription factor EB
TGF-β1: transforming growth factor-β1
TLR9: toll-like receptor 9
TNF-α: tumor necrosis factor-α
TMP: tetramethylpyrazine
ULK1: UNC-52-like kinase 1
UQCRC2: ubiquinol-cytochrome c reductase core protein 2
VDIM: vesicle derived from the inner mitochondrial membrane
